# Nanofocusing with aberration-corrected rotationally parabolic refractive X-ray lenses

**DOI:** 10.1107/S1600577517015272

**Published:** 2018-01-01

**Authors:** Frank Seiboth, Felix Wittwer, Maria Scholz, Maik Kahnt, Martin Seyrich, Andreas Schropp, Ulrich Wagner, Christoph Rau, Jan Garrevoet, Gerald Falkenberg, Christian G. Schroer

**Affiliations:** a Deutsches Elektronen-Synchrotron – DESY, Notkestrasse 85, 22607 Hamburg, Germany; b Linac Coherent Light Source, SLAC National Accelerator Laboratory, 2575 Sand Hill Road, Menlo Park, CA 94025, USA; c Department Physik, Universität Hamburg, Luruper Chaussee 149, 22761 Hamburg, Germany; d Diamond Light Source Ltd, Diamond House, Harwell Science and Innovation Campus, Didcot, Oxfordshire OX11 0DE, UK

**Keywords:** refractive X-ray optics, aberration correction, ptychography, phase plate

## Abstract

Wavefront errors of rotationally parabolic refractive X-ray lenses made of beryllium (Be CRLs) have been recovered for various lens sets and X-ray beam configurations. Due to manufacturing *via* an embossing process, aberrations of individual lenses within the investigated ensemble are very similar. By deriving a mean single-lens deformation for the ensemble, aberrations of any arbitrary lens stack can be predicted from the ensemble with  = 0.034λ. Using these findings the expected focusing performance of current Be CRLs are modeled for relevant X-ray energies and bandwidths and it is shown that a correction of aberrations can be realised without prior lens characterization but simply based on the derived lens deformation. The performance of aberration-corrected Be CRLs is discussed and the applicability of aberration-correction demonstrated over wide X-ray energy ranges.

## Abstract 2

The recovery of wavefront distortions over multiple compound refractive optics was used to model and correct aberrations of similar individual Be lenses for different X-ray energies and focal lengths.

## 1. Introduction   

Compound refractive X-ray lenses (CRLs) (Snigirev *et al.*, 1996 ▸; Lengeler *et al.*, 1998 ▸) made of beryllium (Schroer *et al.*, 2002 ▸) are widely used at synchrotron storage ring sources and X-ray free-electron lasers (XFELs) as beamline optics in order to transport and condition the beam (Chumakov *et al.*, 2000 ▸; Vaughan *et al.*, 2011 ▸; Polikarpov *et al.*, 2014 ▸; Heimann *et al.*, 2016 ▸). Their advantages arise from their large geometric aperture, ranging from 300 µm up to several millimeters, their ability to withstand intense radiation due to a low absorption coefficient and good thermal conductivity, and coherence-preserving quality. In addition, CRLs are in-line optics. Thus, paired with their compact size, they are easy to align and do not alter the beam path when inserted or removed from the beamline.

In order to use these lenses for high-resolution X-ray microscopy, a numerical aperture (NA) greater than 0.5 × 10^−3^ is highly desired. While Be CRLs have been employed for full-field and scanning microscopy applications (Bosak *et al.*, 2010 ▸; Schropp *et al.*, 2013 ▸; Simons *et al.*, 2015 ▸), the unavailabilty of lenses with radii of curvature *R* smaller than 50 µm is a barrier for the highest NAs. Consequently, alternative fabrication techniques like modern lithography and etching were explored to structure high-quality lenses into silicon wafers (Schroer *et al.*, 2003 ▸; Alianelli *et al.*, 2011 ▸; Snigirev *et al.*, 2014 ▸; Patommel *et al.*, 2017 ▸) and polymers (Snigirev *et al.*, 2003 ▸; Nazmov *et al.*, 2004 ▸). Despite their high shape fidelity and material quality, strong absorption in silicon and constraints to a one-dimensional lens shape with small apertures make them mostly applicable at current third-generation synchrotron storage ring sources.

With the increasing availability of XFELs operating in the hard X-ray regime (McNeil & Thompson, 2010 ▸) and the advent of ultra-low-emittance storage rings (Hettel, 2014 ▸), the demand for high-performance refractive optics with large apertures and sufficient radiation hardness is increasing. Accompanying these developments are advances in microstructuring of diamond in order to fabricate one-dimensional lenses by ion/plasma etching (Snigirev *et al.*, 2002 ▸; Nöhammer *et al.*, 2003 ▸; Alianelli *et al.*, 2010 ▸) and one- and two-dimensional lenses by micromachining *via* laser ablation (Polikarpov *et al.*, 2015 ▸; Terentyev *et al.*, 2015 ▸, 2017 ▸). Although rapid progress in shape and surface quality has been made, current diamond lenses do not meet the demands of microscopy with nanometer resolution. Despite mechanical limitations of Be CRLs to smallest curvatures of 50 µm, their superior quality allows for high-resolution microscopy when combining several tens of lenses (Schropp *et al.*, 2015 ▸; Nagler *et al.*, 2016 ▸; Beckwith *et al.*, 2017 ▸). While combining many lenses leads to higher NA, it also increases aberrations due to a superposition of small shape errors. However, it has been demonstrated that residual aberrations can be corrected for by a phase plate (Seiboth *et al.*, 2017 ▸), creating diffraction-limited hard X-ray optics with high NA.

In this article we investigate wavefront distortions of Be CRLs with NA ≥ 0.5 × 10^−3^ for various lens stack combinations, and present an ensemble-wide valid lens shape that describes experiments very well. The gained information is used to compare experimental data with simulations and investigate the influence of aberrations and chromaticity to focal spot properties for relevant lens combinations and photon energies of XFELs and ultra-low-emittance sources. In addition, expected results with aberration-corrected lenses and their applicability over extended photon energy ranges are discussed.

## 2. Lens stack characterization   

**Figure 1:**
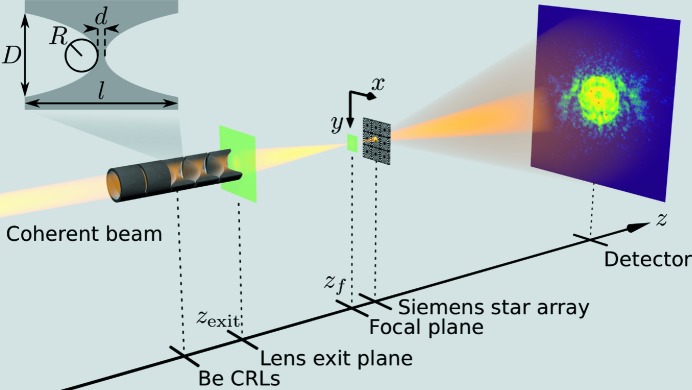
The coherent X-ray beam was focused by a set of *N* Be CRLs onto a Siemens star sample (1 µm-thick tungsten on a diamond membrane). The sample was scanned in a grid fashion in the *x* and *y* direction. We collected a diffraction pattern for every scan point in order to reconstruct the complex-valued probe function *via* ptychography. The inset shows the geometry for a single lens with geometric aperture *D*, radius of curvature *R*, lens thickness *l* and distance between parabola apices *d*.

Both the available raw Be materials and mechanical limitations while pressing the lens shape into the Be foil limit feasible lens apertures *D* and radii of curvatures *R* (see inset in Fig. 1 ▸). Here, the Be material IF-1 (Materion) is used due to its superior purity and X-ray optical quality (Roth *et al.*, 2014 ▸). Unfortunately, the foil thickness *l* is limited to 500 µm. Since *R* is one of the most crucial parameters for building refractive optics with high NA, lenses with the smallest available curvature 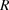 = 50 µm are used exclusively. Given these constraints and the lens geometry (*cf*. inset in Fig. 1 ▸), the geometric aperture 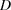 = 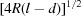 is 306 µm with a typical distance between the parabola apices of 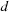 = 30 µm. In the experiments an aperture-defining pinhole with 300 µm diameter was used to only expose the coined lens to incoming X-rays. Experiments were carried out at beamline P06 of PETRA III at DESY (Schroer *et al.*, 2016 ▸) and beamline I13 at the Diamond Light Source (DLS) (Rau *et al.*, 2010 ▸). We characterized lens stacks with a varying number of lenses *N* at different photon energies *E* as shown in Table 1 ▸.

For both synchrotron radiation facilities the full-width at half-maximum (FWHM) horizontal undulator source size 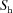 is too large to achieve a sufficient FWHM transverse coherence length in the horizontal direction 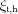 = 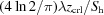 at the main optics position 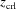 downstream of the undulator source. For coherent experiments the transverse coherence length should ideally exceed the optics aperture *D*. At P06 the default 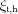 without any beamline optics ranges from 130 µm to 90 µm for 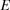 = 8.2 keV to 12 keV, respectively. At I13 the coherence length 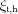 ≃ 80 µm for 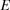 = 8.2 keV. Therefore, we created a secondary source at both facilities, using the available optics at the beamlines. At P06 we used 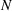 = 2 two-dimensional Be CRLs with 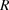 = 50 µm positioned 43.5 m downstream of the undulator source to create a small secondary source. At I13 we used the horizontal front-end slits located 13 m downstream of the undulator source closed down to 50 µm as a horizontal source. The achieved coherence lengths 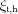 can be found in Table 1 ▸. The lateral coherence length in the vertical direction is sufficient at both light sources and therefore omitted here. The slightly different source position in the horizontal and vertical direction at I13 is negligible due to the very long beamline layout of 250 m and leads to changes in the image distance of the main optics of only 20 µm. In both cases the beam divergence leads to an overfilling of the optics aperture. For I13 the beam size is 12 mm × 6 mm and for P06 the beam size ranges from 7.7 mm × 3.2 mm down to 3.2 mm × 1.4 mm (h × v) for photon energies from 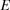 = 8.2 keV to 12 keV. Compared with the circular aperture of the main CRLs with 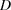 = 300 µm the optics are well overfilled in each case.

**Figure 2:**
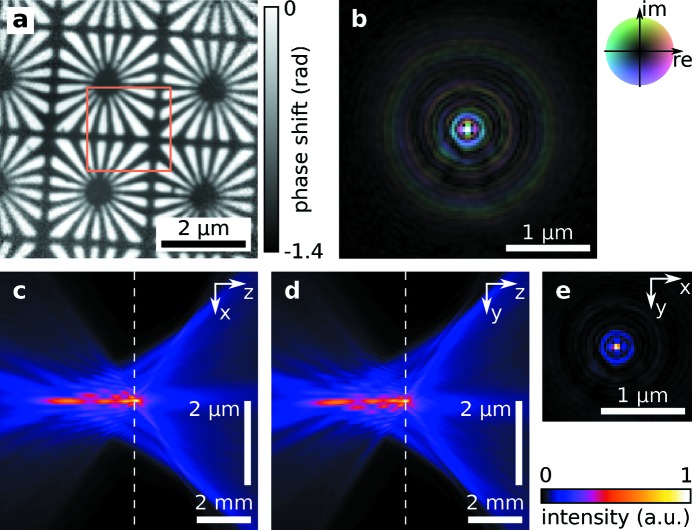
The reconstructed object phase shift (*a*) and the recovered complex probe function in the sample plane with 2× enhanced contrast (*b*) is shown exemplarily for 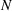 = 50 Be CRLs at I13 (*cf*. Table 1 ▸). With the probe function the X-ray beam caustic in the horizontal direction (*c*) and vertical direction (*d*) was calculated, showing clear indication of spherical aberration. The intensity distribution in the focal plane, marked by the dashed line in both (*c*) and (*d*), is shown in (*e*).

To measure lens aberrations we characterized the wavefront after the lens near its focal plane using ptychography (Thibault *et al.*, 2008 ▸; Kewish *et al.*, 2010 ▸; Schropp *et al.*, 2010 ▸; Hönig *et al.*, 2011 ▸; Vila-Comamala *et al.*, 2011 ▸). A test sample, here a Siemens star array, was placed in the vicinity of the focal plane, typically within 1 mm. The beam is scanned over the sample with overlap between neighboring scan points in a grid fashion with a step size of 100 nm as shown in Fig. 1 ▸. Typically an area of 2 µm × 2 µm was scanned [orange box in Fig. 2(*a*) ▸], leading to 441 diffraction patterns. From the diffraction patterns recorded at known positions the algorithm reconstructs both the complex-valued transmission function of the object and complex-valued probe function (Thibault *et al.*, 2008 ▸). Figs. 2(*a*) and 2(*b*) ▸ show the phase shift of the reconstructed object and the complex-valued wavefield of the retrieved probe for 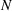 = 50 Be CRLs measured at I13 (*cf*. Table 1 ▸), respectively. From this complex probe function, the wavefield can be propagated to any position behind the lens using the Fresnel–Kirchhoff diffraction integral, as described by Born & Wolf (1980 ▸; ch. 8.3.2). In this way the beam caustic in the horizontal (Fig. 2*c* ▸) and vertical projection (Fig. 2*d* ▸) was calculated. To extract focal spot properties, the wavefield was propagated to the focal plane 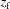, defined by the peak intensity on the optical axis [Fig. 2(*e*) ▸ and dashed lines in Figs. 2(*c*) and 2(*d*) ▸]. To characterize the lens shape and design appropriate corrective phase plates (Seiboth *et al.*, 2017 ▸), the wavefield was propagated to the lens exit 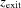. By analysing the distorted wavefront the strength of various aberrations for each lens stack was determined (Seiboth *et al.*, 2016 ▸) and consecutively used to model lens deformations.

## 3. Single-lens deformation   

While the measured wavefront is linked to the individual lens stack, each stack consists of many single lenses that are manufactured by a mechanical coining process using the same stamping tool for each lens. In the experiments all stacks were assembled from the same ensemble, consisting of 50 single lenses. The Be IF-1 lenses were acquired from RXOPTICS between 2011 and 2016. To investigate whether all lenses can be described with similar lens deformation, we first iteratively refined the lens shape for each experiment. Based on knowledge about the fabrication process, we assume rotational symmetry around the optical axis for the lens shape. The overall shape is described by a parabola, deformations are modeled by a set of four Gaussian distributions with variable radical position, width and amplitude. The functions were chosen to best match the dominant spherical aberration and a common pattern of phase errors across all experiments. We found that using only spherical Zernike polynomials to describe the lens deformation required more coefficients to sufficiently describe the deformation, especially near the center of the lens.

**Figure 3:**
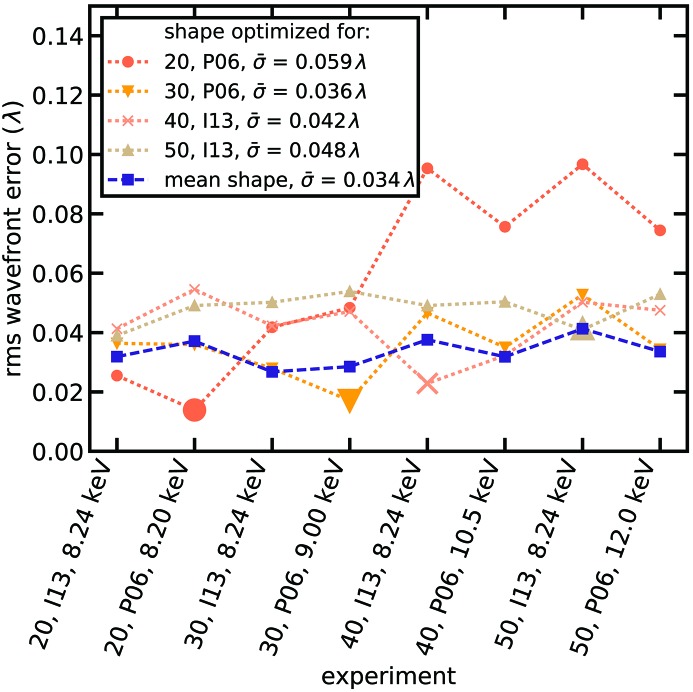
The deformation of a single lens was modeled to best fit the data from the corresponding experiment (enlarged circle, down-triangle, cross and up-triangle). The same lens shape was then also applied to model all other experiments. The dotted line with circles shows the lens shape optimized for 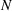 = 20 lenses at beamline P06 (*cf.* Table 1 ▸). The dotted lines with down-triangle, cross and up-triangle show results for 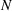 = 30 lenses at P06, 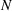 = 40 lenses at I13 and 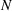 = 50 lenses at I13, respectively. The dashed line with squares shows the result when using a mean deformation over all experiments. The mean error 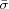 for the given shape over all experiments is noted in the legend.

Assuming a spherical wave from the undulator source falling onto the optics aperture, the wavefield was propagated to the first lens in the stack. When applying the thin lens approximation for every single lens in the stack, the wavefield can be multiplied by the complex-valued transmission function of the single lens. The resulting wavefield is propagated to the next lens in the stack and the transmission function is applied again. This process continues until the end of the stack is reached. By comparing the resulting wavefield with experiments, an iterative refinement of the lens deformation was carried out. After only a few iteration steps (<5), good agreements for the individual experiments could be achieved, exemplarily shown by the enlarged circle, down-triangle, cross and up-triangle in Fig. 3 ▸. Applying the same shape to other configurations, however, we observed a stronger disagreement with experiments in most cases (*e.g.* the orange dotted line with circles in Fig. 3 ▸). As the lenses for each experiment were assembled randomly, some variations are expected. In the case of 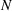 = 20 lenses (orange dotted line with circles in Fig. 3 ▸) the agreement with experiments using up to 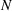 = 30 lenses is good, but for the largest lens stacks the error increases. We believe that the small lens stack might have contained a large number of lenses with a deformation slightly different from the average. Therefore, a larger error is observed when using most of the lenses in the largest stacks due to averaging.

**Figure 4:**
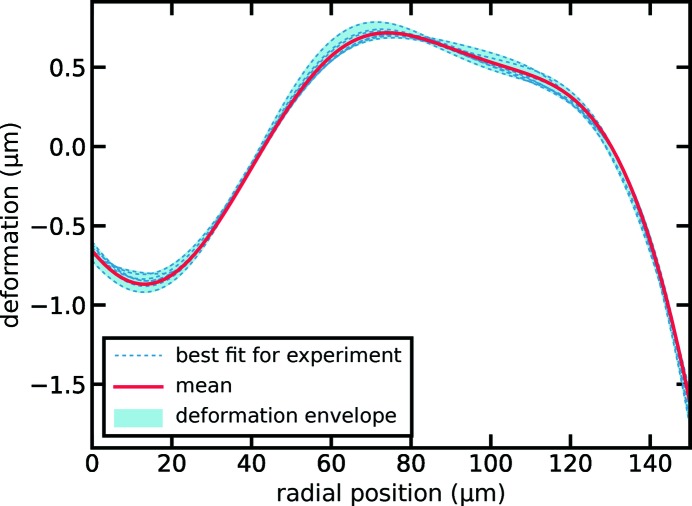
The shape deviation from a perfect paraboloid of rotation with radius of curvature 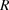 = 50 µm for a single lens surface is shown over the distance from the optical axis. Refined shapes for individual experiments are depicted by the dashed blue lines. The envelope for all shapes is shown by the light blue area. The mean deformation over all experiments is represented by the solid red line.

By plotting the retrieved shape deformation for all experiments in Fig. 4 ▸ (dashed lines and envelope) only minor differences in lens shape were revealed. Using a mean deformation over all experiments (solid red line in Fig. 4 ▸) we reached very good agreement with a mean standard deviation of 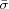 = 0.034λ across all experiments (*cf*. dashed blue line with squares in Fig. 3 ▸). In the following we will use this mean deformation to discuss focusing properties for a variety of lens configurations for relevant X-ray energies and bandwidths.

## 4. Focusing properties and aberration correction   

**Figure 5:**
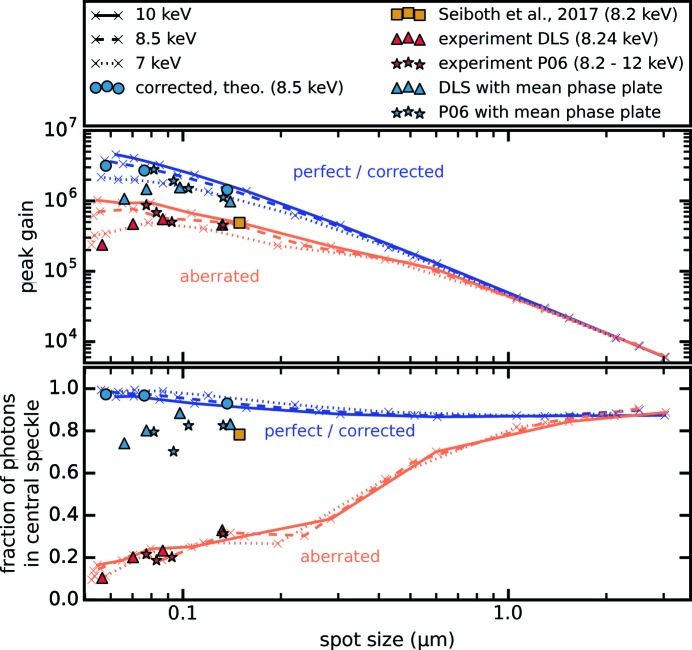
The focusing performance in terms of peak gain and fraction of photons in the central speckle is shown *versus* the focal spot size, achieved by combining 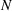 = 1 to 60 lenses with 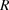 = 50 µm. Aberrated lenses (using the mean deformation from Fig. 4 ▸) for 10, 8.5 and 7 keV are shown by the orange solid, dashed and dotted lines, respectively. Associated experimental data are shown by the red triangles and stars for I13 and P06, respectively. The theoretically achievable performance with a perfect phase plate fitted after the lens stack, that is, no remaining wavefront errors, and taking phase plate absorption into account, is shown by the blue circles. Experimental results from Seiboth *et al.* (2017 ▸) are represented by the yellow square. Aberration-free lenses for 10, 8.5 and 7 keV are shown by the blue solid, dashed and dotted line, respectively. Calculated results for applying a phase plate derived from the mean lens shape (*cf*. Fig. 4 ▸) to the DLS and P06 experimental data are represented by the blue triangles and stars, respectively.

To investigate the deterioration of focusing performance for a variety of Be CRL stacks and photon energies under the assumption of the calculated mean shape (*cf*. Fig. 4 ▸), we assessed the peak gain and fraction of photons within the central speckle for stacks of 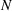 = 1 to 60 Be CRLs, shown in Fig. 5 ▸. The focal spot size 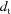 is given by (Schroer *et al.*, 2013 ▸)
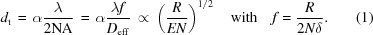
Here, α is a geometry factor of the aperture shape, 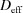 is the effective optics aperture reduced due to absorption in the lens, *E* is the X-ray energy and δ is the refractive index decrement. The peak gain 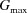 is defined as the ratio between maximum intensity 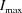 within the focal plane of a given optical system compared with the flat beam intensity 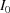 on the optics aperture:
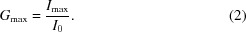
Since reconstructions and numerical simulations have a discrete pixel size *p*, we determine the peak intensity in the focal plane 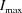 = 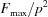 *via* the pixel with highest photon flux 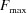. The flat beam intensity 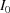 = 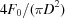 is given by the initial photon flux 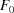 within the optics aperture. Transmission of the lens stack and optionally of the phase plate is accounted for within 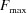. Numerical calculations were carried out with a pixel size of 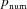 = 20 nm. The pixel size 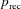 in the various experiments varied and is summarized in Table 1 ▸. We determined the FWHM focal spot size 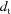 by fitting a two-dimensional Gaussian to the central speckle of the focal plane. For the fraction of photons within the central speckle we considered values within 3σ of the Gaussian fit.

When modeling aberration-free lenses (blue lines in Fig. 5 ▸) the peak gain scales inversely quadratic with the focal spot size and is only dampened by the increased absorption in larger lens stacks, eventually reaching a maximum at spot sizes below 60 nm when the reduction in focus size is offset by additional absorption. For lower photon energies this is more severe due to a higher absorption coefficient. The fraction of photons in the central speckle increases slightly for smaller spot sizes. When more and more lenses are stacked, the aperture changes gradually from a flat circular aperture (negligible absorption in the outermost lens part), over a Gaussian truncated by the geometric aperture *D*, into a Gaussian profile completely dominated by strong absorption in the outermost lens part due to the concave parabolic lens shape. As the focal spot is the Fourier transform of the aperture function, side lobes reduce when going from an Airy disk profile to a Gaussian focal spot.

With increasing NA of aberrated lenses (orange lines in Fig. 5 ▸) their focusing performance deteriorates. As more lenses are required for smaller focal spots, wavefront errors add up. They scale linearly with the number *N* of lenses when neglecting beam convergence within the lens stack. Therefore, the gain is hardly influenced for micrometer-sized spots, but is considerably more dampened with increasing NA. For higher X-ray energies of several tens of keV a similar deterioration with numerical aperture can be observed. Although considerably more lenses are required to reach the same focal spot size, the strength of spherical aberration is comparable. As the refractive index decrement δ scales with 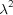 in the hard X-ray range, many more lenses are necessary to achieve a similar wavefront deformation. The strong spherical aberration for optics with spot sizes in the nanometer range dramatically reduces the fraction of photons within the central speckle of the focus and thus also influences the gain. The optical performance of measured CRL stacks (red stars and triangles in Fig. 5 ▸) agrees very well with numerical calculations (orange lines in Fig. 5 ▸).

In the past the correction of residual aberrations in a stack of 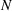 = 20 Be CRLs, after measuring wavefront errors for the given lens stack, was demonstrated by Seiboth *et al.* (2017 ▸) using a fused silica phase plate. The corresponding data are represented by the yellow square in Fig. 5 ▸. A strong increase in the fraction of photons within the central speckle was observed, indicating a nearly diffraction-limited optical system. The peak gain is significantly influenced by absorption due to a very thick phase plate substrate used by the authors. However, simulations show that the performance of a perfect system can almost be restored when using fused silica phase plates with optimized substrate thickness, that are specifically designed for the corresponding optics (blue circles in Fig. 5 ▸), *i.e.* a perfect match between lens aberrations and phase plate correction. When modeling the corrected lens stacks by a fused silica phase plate based upon the calculated mean shape, residual wavefront errors remain (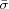 = 0.034λ) and performance is slightly reduced (blue stars and triangles in Fig. 5 ▸).

## 5. Influence of chromaticity   

**Figure 6:**
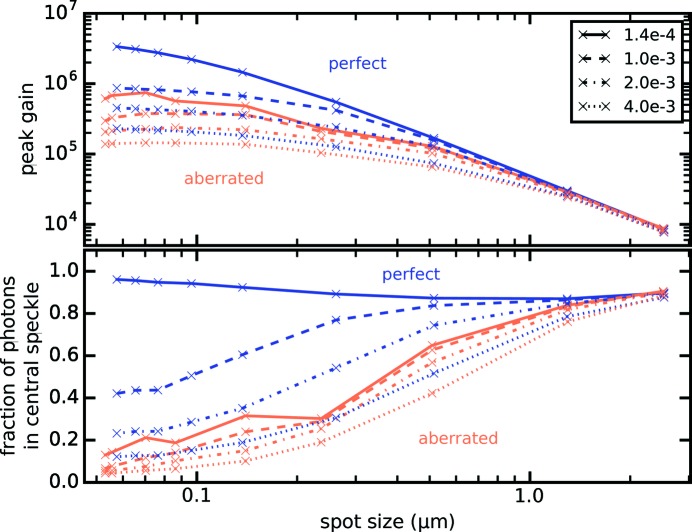
The focusing performance in terms of peak gain and fraction of photons in the central speckle *versus* the focal spot size is assessed at a mean X-ray photon energy of 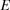 = 8.5 keV for different X-ray bandwidths, assuming a constant integral energy. 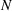 = 1 to 60 lenses with 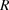 = 50 µm were used to model the data, using a perfect lens shape (blue lines) and the mean deformation (orange lines) as shown in Fig. 4 ▸.

As refractive optics, Be CRLs naturally exhibit chromatic aberration. The change of focal length with a change of X-ray energy leads to a broadening of the focal spot profile and an increase in side lobe intensity for wide bandwidths, which is especially relevant for applications at XFELs operating in self-amplified spontaneous emission (SASE) mode (Seiboth *et al.*, 2014 ▸). Effects on focusing performance for X-ray bandwidths typical of SASE operation are shown in Fig. 6 ▸ for 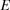 = 8.5 keV and 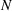 = 1 to 60 lenses. Calculations assume a constant integral energy for all four investigated bandwidths. Following Seiboth *et al.* (2014 ▸), we convolved the monochromatic three-dimensional intensity distribution with the spectral distribution mapped to a change in focal length on the optical axis. This assumes that the wavefront shape is not significantly altered for the mild polychromaticity here, but the focal plane simply shifts along the optical axis while the transverse profile is conserved.

For a narrow bandwidth of 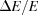 = 1.4 × 10^−4^, *e.g.* by a Si-111 monochromator, or for a seeded XFEL, the influence on focusing properties is negligible, as the change in focal length is considerably smaller than the depth-of-field in this case. Aberrated lenses suffer less from an increasing bandwidth, as the focal spot already shows strong side lobes which are only slightly enhanced by the shifting focal plane. On the other hand diffraction-limited lenses are heavily influenced, as the tight Gaussian focal spot becomes deteriorated by an increasingly strong background when the bandwidth increases [*cf*. Fig. 6 ▸ and Seiboth *et al.* (2014 ▸)]. When aiming for highest intensities, *i.e.* combining 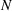 = 60 lenses for best gain, the reachable peak intensities for a perfect lens are reduced by a factor of 3.9, 7.5 and 14.6 when going from 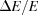 = 1.4 × 10^−4^ to X-ray bandwidths 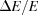 of 1 × 10^−3^, 2 × 10^−3^ and 4 × 10^−3^, respectively. In comparison, the lens with spherical aberration only loses a factor of 2.1, 3.0 and 4.4 relative to the peak intensity in the monochromatic aberrated case. Since the latter is reduced by a factor of 5.5 compared with the aberration-free lens, the peak intensity is in all cases smaller than that of the ideal optics. The peak intensity can be increased by a factor of 5 for a monochromatic beam and by a factor of roughly 2 for SASE when correcting an aberrated Be CRL with NA ≥ 0.5 × 10^−3^.

Depending on the available pulse energy and length for any given bandwidth, a broadband SASE pulse might achieve higher peak intensities than a seeded beam, or *vice versa*. However, in all scenarios diffraction-limited optics will deliver a cleaner focus with fewer photons in side lobes or as background compared with the aberrated one.

## 6. Aberration correction over broad X-ray energy ranges   

**Figure 7:**
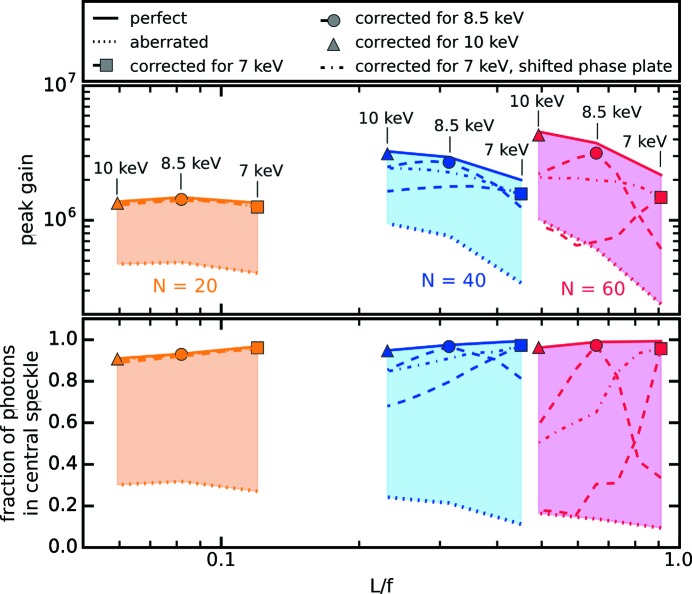
Focusing performance for lens stacks with 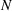 = 20, 40 and 60 lenses as a function of lens length *L* over focal length *f*. Solid lines mark the best achievable performance of an aberration-free lens. Dotted lines represent lenses affected by spherical aberration. The triangle, circle and square show results with phase plates designed for 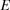 = 10 keV, 8.5 keV and 7 keV, respectively. Dashed lines indicate the performance if the phase plate is used at differing energies than the design one. The dash-dotted line indicates the performance of a phase plate designed for 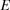 = 7 keV that is positioned further downstream for higher energies according to the change in focal length.

Corrective phase plates rectify wavefront errors by inducing a phase shift that offsets the wavefront distortion. Thus, they need to be fitted in terms of their geometric shape to the particular experiment. This means a specifically designed phase plate for each individual lens stack and X-ray energy to achieve the best performance. However, the data shown in Fig. 3 ▸ suggest that a very high degree of correction (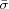 = 0.034λ) can be achieved by designing a phase plate based on a widely applicable mean shape. Additionally, the refractive index decrement δ scales with 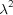 in the hard X-ray range, independent of the material. Both the induced phase error in the lens and the corrective phase shift of the phase plate scale in the same way with varying energy and cancel each other. Thus, phase plates should work over a very wide X-ray energy range for refractive optics. However, this is only valid if the qualitative shape of wavefront deformations stays constant over the whole energy range. For a thin lens, where the ratio 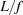 of lens length 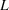 = 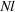 (*cf*. Fig. 1 ▸) and focal length *f* is 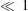, this is true. In the case of a thin lens, the wavefield does not converge significantly within the lens stack. Thus, since every lens is deformed in the same way, the shape of the wavefront distortion is preserved, only scaling in magnitude with *N*. Fig. 7 ▸ depicts this case for 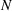 = 20 (orange graphics), where 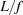 ≤ 0.1. Phase-plate-corrected thin optics achieve nearly aberration-free performance for all energies, even when the phase plates are used at differing energies (dashed and dash-dotted lines in Fig. 7 ▸).

For thick lenses the focal length *f* is about the same as the overall lens length *L*, that is, 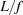 ≃ 1. In this case the beam converges significantly within the lens stack. As the beam size reduces within the stack, consecutive single lenses are only illuminated in parts. Thus, the wavefront is only affected by parts of the shape deformation of the lens. The wavefront error does not simply scale in magnitude with *N*, but the shape of the wavefront deformation is altered gradually. This case is shown in Fig. 7 ▸ for 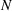 = 40 and 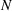 = 60 lenses by the blue and red graphics, respectively. The performance of aberration-free optics can again be restored by corrective phase plates (triangle, circle and square). As thicker lens stacks induce more wavefront errors, they require thicker phase plates to correct for. Absorption of the fused silica phase plate becomes significant, especially at lower X-ray energies (*cf*. squares at 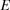 = 7 keV). Changing the X-ray energy while using the same phase plates results in reduced performance (dashed lines), becoming more severe with increasing 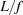.

To counterbalance these effects to some extent, the phase plate can be shifted along the optical axis (dash-dotted line) by a distance 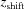. The idea is to match at least the diameter of the phase plate to the diameter of the X-ray beam, although the phase plate shape will be slightly off. The propagation of a ray hitting the entrance of a thick lens stack (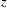 = 0) at a distance 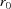 from the optical axis can be approximated by a sinusoidal path inside the stack (Patommel, 2010 ▸; Schroer *et al.*, 2013 ▸),
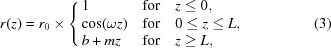
with 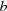 = 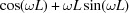 and 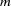 = 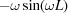. The convergence is determined by the refractive power of the lens 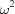 = 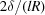. Aperture rays (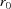 = *D*/2) exit the lens stack at 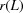 = 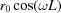 with a slope 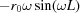. As ω scales with λ we can compensate a change in convergence inside the lens stack at an X-ray energy 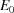 by a phase plate shift 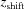 at energy 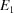 with
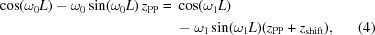
and 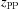 being the phase plate position relative to the lens exit at 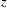 = 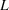. In cases where the phase plate cannot be positioned within the lens stack, 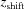 ≥ 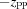. When the phase plate is designed to be positioned directly after the lens stack (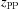 = 0) at 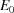, only energy changes 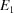 > 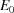 can be corrected for (dash-dotted lines for 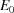 = 7 keV in Fig. 7 ▸). If the initial phase plate design at 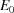 is based on a position further downstream of the lens (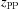 > 0) a correction can also be applied for energies 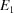 < 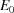.

## 7. Conclusion   

A refinement of the lens deformation over eight individual experiments allowed us to determine a mean value of Be CRL deformations that results in very good agreement when compared with experimental data. The finding allows modeling of IF-1 Be CRL stacks with 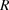 = 50 µm for any number of lenses at variable photon energies. This enables one to calculate wavefront aberrations caused by the optics with high fidelity (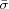 = 0.034λ). At the same time a reduction of aberrations as shown by Seiboth *et al.* (2017 ▸) can be achieved without any additional lens characterization for any combination of lenses from the ensemble. A corrective phase plate can be shaped from numerical simulations alone and reduce wavefront aberrations for any lens stack down to 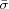 = 0.034λ. Results show that lens deformations are negligible in focusing performance for spot sizes 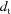 ≥ 1 µm (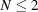 for the X-ray energies considered here). For larger lens stacks with 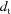 ≤ 200 nm the spherical aberration degrades the focus severely and bandwidth effects, *i.e.* when operating in SASE mode, have a significant influence. When correcting larger Be CRL stacks with a phase plate the usability of the phase plate over a larger energy range depends on the ratio 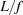, which describes the lens thickness. For stacks with 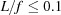 a corrective phase plate can be used over energy ranges of several keV. Phase plates for thicker lens stacks are more susceptible to changes in beam convergence inside the lens stack. It was found that the effect can be compensated in part by a shift of the phase plate along the optical axis to increase the energy range. The broad availability of nanofocusing X-ray optics with large apertures and sufficient radiation hardness is crucial for applications at XFELs and ultra-low-emittance storage rings. The present work suggests a method to improve the performance of Be CRL optics across a wide range of instruments to provide diffraction-limited optics without the need of prior on-site metrology.

## Figures and Tables

**Table 1:** Lens configuration and beamline (BL) properties (photon energy *E* and lateral coherence length in horizontal direction 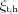) for all experiments For all lenses, 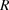 = 50 µm and 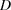 = 300 µm. Focal length *f* and NA are theoretical values. The full width at half-maximum focal spot size 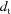 was determined from the reconstructed wavefield (pixel size 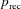) in the focal plane. The focal spot size with phase plate 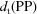 is numerically modeled based on using a phase plate derived from the found mean deformation (*cf*. Fig. 4 ▸).

*N*	BL	*E* (kev)	ξ_t,h_ (µm)	*f* (mm)	NA (×10^−3^)	*d*_t_ (nm)	*d*_t_(PP) (nm)	*p*_rec_ (nm)
20	P06	8.20	1120	251	0.49	133	133	29.3
20	I13	8.24	630	253	0.48	132	140	31.4
30	P06	9.00	830	204	0.58	92	104	26.7
30	I13	8.20	630	170	0.66	87	98	31.8
40	P06	10.50	490	210	0.57	83	94	22.9
40	I13	8.20	630	131	0.78	70	77	32.1
50	P06	12.00	310	221	0.55	77	81	20.0
50	I13	8.20	630	109	0.88	56	66	32.2
