# Nanofocusing with aberration-corrected rotationally parabolic refractive X-ray lenses. Corrigendum

**DOI:** 10.1107/S1600577521003167

**Published:** 2021-05-01

**Authors:** Frank Seiboth, Felix Wittwer, Maria Scholz, Maik Kahnt, Martin Seyrich, Andreas Schropp, Ulrich Wagner, Christoph Rau, Jan Garrevoet, Gerald Falkenberg, Christian G. Schroer

**Affiliations:** a Deutsches Elektronen-Synchrotron – DESY, Notkestrasse 85, 22607 Hamburg, Germany; b Linac Coherent Light Source, SLAC National Accelerator Laboratory, 2575 Sand Hill Road, Menlo Park, CA 94025, USA; c Department Physik, Universität Hamburg, Luruper Chaussee 149, 22761 Hamburg, Germany; d Diamond Light Source Ltd, Diamond House, Harwell Science and Innovation Campus, Didcot, Oxfordshire OX11 0DE, United Kingdom

**Keywords:** refractive X-ray optics, aberration correction, ptychography, phase plate

## Abstract

A correction in the paper by Seiboth *et al.* [(2018). *J. Synchrotron Rad.***25**, 108–115] is made.

In the paper by Seiboth *et al.* (2018) ▸, there is an error in the caption of Fig. 4. In the original paper, it is stated that the depicted lens deformation is ‘for a single lens surface’. However, the shown error is for a single lens, which is a bi-concave lens. Thus, the depicted error appears by a factor of 2 larger. In the body of the original article and all other occurrences, especially in Section 3, we refer to the error of a ‘single lens’ instead of a ‘single lens surface’, which is correct.

The correct caption is shown along with the figure below. ▸

## Figures and Tables

**Figure 4:**
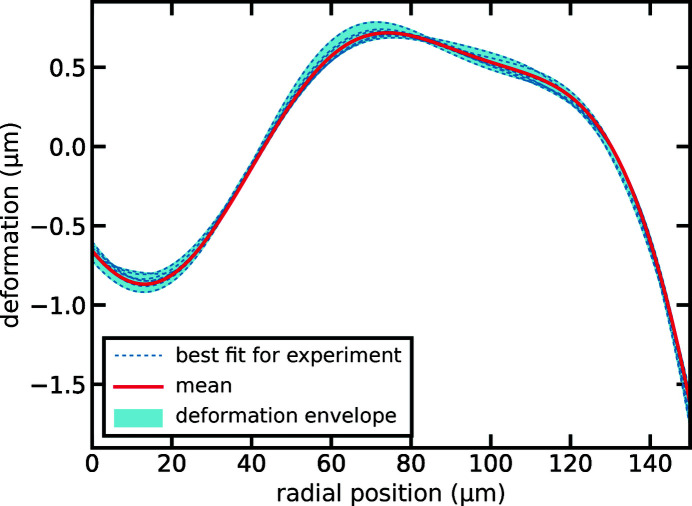
The shape deviation from a perfect paraboloid of rotation with radius of curvature *R* = 50 µm for a single lens is shown over the distance from the optical axis. Refined shapes for individual experiments are depicted by the dashed blue lines. The envelope for all shapes is shown by the light blue area. The mean deformation over all experiments is represented by the solid red line.
